# Genome-wide transcription start site profiling in biofilm-grown *Burkholderia cenocepacia* J2315

**DOI:** 10.1186/s12864-015-1993-3

**Published:** 2015-10-13

**Authors:** Andrea M. Sass, Heleen Van Acker, Konrad U. Förstner, Filip Van Nieuwerburgh, Dieter Deforce, Jörg Vogel, Tom Coenye

**Affiliations:** Laboratory of Pharmaceutical Microbiology, Ghent University, Ottergemsesteenweg 460, 9000 Ghent, Belgium; Laboratory of Pharmaceutical Biotechnology, Ghent University, Ghent, Belgium; Core Unit Systems Medicine, University of Würzburg, Würzburg, Germany; Institute for Molecular Infection Biology, University of Würzburg, Würzburg, Germany

**Keywords:** *Burkholderia cenocepacia*, Biofilms, dRNA-Seq, Transcription start site, Small RNAs, Antisense RNA, Genomic islands

## Abstract

**Background:**

*Burkholderia cenocepacia* is a soil-dwelling Gram-negative Betaproteobacterium with an important role as opportunistic pathogen in humans. Infections with *B. cenocepacia* are very difficult to treat due to their high intrinsic resistance to most antibiotics. Biofilm formation further adds to their antibiotic resistance. *B. cenocepacia* harbours a large, multi-replicon genome with a high GC-content, the reference genome of strain J2315 includes 7374 annotated genes. This study aims to annotate transcription start sites and identify novel transcripts on a whole genome scale.

**Methods:**

RNA extracted from *B. cenocepacia* J2315 biofilms was analysed by differential RNA-sequencing and the resulting dataset compared to data derived from conventional, global RNA-sequencing. Transcription start sites were annotated and further analysed according to their position relative to annotated genes.

**Results:**

Four thousand ten transcription start sites were mapped over the whole *B. cenocepacia* genome and the primary transcription start site of 2089 genes expressed in *B. cenocepacia* biofilms were defined. For 64 genes a start codon alternative to the annotated one was proposed. Substantial antisense transcription for 105 genes and two novel protein coding sequences were identified. The distribution of internal transcription start sites can be used to identify genomic islands in *B. cenocepacia*. A potassium pump strongly induced only under biofilm conditions was found and 15 non-coding small RNAs highly expressed in biofilms were discovered.

**Conclusions:**

Mapping transcription start sites across the *B. cenocepacia* genome added relevant information to the J2315 annotation. Genes and novel regulatory RNAs putatively involved in *B. cenocepacia* biofilm formation were identified. These findings will help in understanding regulation of *B. cenocepacia* biofilm formation.

**Electronic supplementary material:**

The online version of this article (doi:10.1186/s12864-015-1993-3) contains supplementary material, which is available to authorized users.

## Background

*Burkholderia cenocepacia* J2315 is a member of the *Burkholderia cepacia* complex (Bcc), a group of 18 species of closely related Gram-negative Betaproteobacteria [1] which occur in the soil rhizosphere and also play an important role as opportunistic pathogens in humans [2–4]. Bcc bacteria are intrinsically resistant to most antibiotics, and infections with Bcc bacteria are therefore difficult to treat. Bcc bacteria are also able to form biofilms, further adding to their recalcitrance to antibiotic treatment [4].

*B. cenocepacia* J2315 harbours a large 8.06 Mb multi-replicon genome with a high average GC-content of 66.9 %. The genome consists of two large replicons of 3.87 and 3.22 Mb, a smaller replicon 0.88 Mb and a plasmid 0.09 Mb in size, with 7261 annotated protein coding and 113 annotated RNA genes [5], including 74 tRNAs and 10 riboswitches. However, transcription start sites (TSS), 5′ untranslated regions (5′UTRs) of annotated genes and regulatory non-coding small RNAs have not yet been comprehensively analysed and annotated. Emerging new RNA sequencing techniques, notably differential RNA sequencing (dRNA-Seq, [6]), make it now possible to precisely map the transcription start sites over a whole genome, and at the same time discover novel genome features.

Primary transcripts of prokaryotes carry a triphosphate at their 5′-end, whereas 5′-ends derived from processing and degradation carry a monophosphate. The dRNA-Seq approach uses the properties of a 5′-monophosphate-dependent exonuclease (Terminator™ 5′-Phosphate-Dependent Exonuclease, TEX) to selectively degrade processed transcripts, thereby enriching for un-processed RNA species carrying a native 5′-triphosphate. TSS can then be identified by comparing TEX-treated with untreated RNA-seq libraries, as they appear as localised maxima in coverage enriched by TEX-treatment [6].

dRNA-Seq enables precise mapping of 5′ ends of transcripts, whereas coverage over the whole transcript length is usually poor and 3′ end of transcripts are only represented for short transcripts. For this reason the dRNA-Seq datasets were compared to conventional global RNA-seq data (gRNA-Seq) which provide more even coverage and a more comprehensive representation of full length transcription units. This approach aids in evaluating the function of an identified TSS, particularly for TSS internal to genes.

The aim of the present study is to identify genes expressed in *B. cenocepacia* biofilms and detect the regulatory elements that might be involved in biofilm formation and survival, as a prerequisite to develop new strategies in treatment of *B. cenocepacia* infections.

## Results and discussion

### TSS annotation

**Fig. 1:**
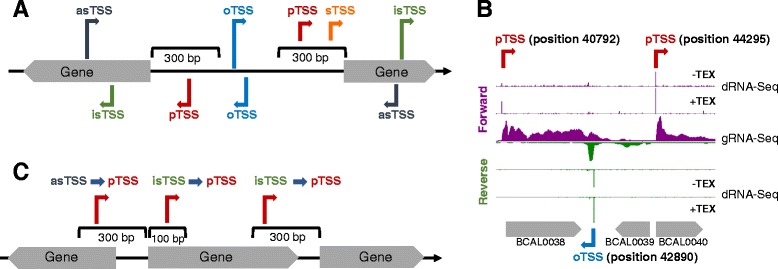
Categorisation of transcription start sites. Panel **a**: Initial TSS categorisation into primary (pTSS), secondary (sTSS), internal sense (isTSS) and internal antisense (asTSS) based on differential RNA sequencing. Panel **b**: Comparison of dRNA-Seq and gRNA-Seq data for genes BCAL0038-0040, visualised with the Integrated Genome Browser [51]. dRNA-Seq data are represented as read starts per base, with a vertical scale of 100 for forward reads and 7000 for reverse reads. gRNA-Seq data are represented as coverage, with a vertical scale of 500 for forward reads and 1000 for reverse reads. Panel **c**: Re-categorisation of internal TSS guided by leading edges of transcription identified by global RNA sequencing

dRNA-Seq of duplicate biofilm-derived RNA samples resulted in datasets with 2.4–4.1 million mapped reads, gRNA-Seq of triplicate biofilm-derived RNA samples resulted in datasets with 23–33 million mapped reads (Additional file 1: Table S1). A total of 10843 TSS were automatically annotated based on the dRNA-Seq data (Additional file 2: Table S2), evenly distributed on forward and reverse strands. 3908 TSS remained after noise filtering on a minimum of 10 read starts (Table 1, Additional file 3: Table S3). These were then categorised according to their position in relation to annotated genes (Fig. 1a): TSS in intergenic regions, located ≤ 300 nt upstream of the start of and in sense with an annotated gene, were assigned primary TSS (pTSS) for the respective gene. TSS internal to annotated genes were assigned internal sense (isTSS) or antisense (asTSS). TSS in intergenic regions and not associated with any gene were assigned “orphan” (oTSS). Where TSS were positioned within 100 nt of and same sense to a primary or orphan TSS, they were designated secondary (sTSS).

**Table 1:** Number of transcription start sites by category

	Replicon 1	Replicon 2	Relicon 3	Plasmid	Total
Genes	3622	2859	781	100	7374
TSS	6815	3010	914	104	10843
Total categorised TSS	2595	1142	316	57	4010
pTSS	1271	671	136	11	2089
depleted pTSS	64	24	8	6	102
pTSS internal same gene	42	27	2	1	72
pTSS internal upstream gene	78	22	5	0	105
oTSS	237	126	57	8	428
sTSS	181	61	19	0	261
isTSS	502	140	55	15	712
asTSS	304	144	49	23	520

TSS loci were also compared to the global transcriptome datasets by manual inspection. More than 90 % of intergenic pTSS were accompanied by an abrupt increase in coverage in the gRNA-Seq dataset (Fig. 1b), substantiating that they are *bona fide* loci for transcription initiation. For the purpose of differentiation from TSS based on dRNA-Seq data, we designate these abrupt increases in gRNA-Seq data coverage “leading edges of transcription” (LEs, [7]) for the rest of the manuscript.

LEs found internal to genes were used to assign function to internal TSS: where internal TSS were associated with LEs and positioned ≤300 nt upstream or ≤100 nt downstream and in sense with a gene lacking an intergenic pTSS, they were re-assigned pTSS for the respective gene (Fig. 1c).

Transcription can be primed by molecules other than NTPs, e.g. nano rRNAs [8]. The resulting primary transcripts do not carry a triphosphate at their 5′-end and are depleted by TEX-treatment. Where depleted local read start maxima coincided with a distinct LE, they were reported as depleted pTSS (Table 1, Additional file 3: Table S3), adding another 102 pTSS to the dataset.

In total 2089 pTSS were annotated over the whole *B. cenocepacia* genome for genes transcribed under biofilm conditions, representing 28 % of all annotated genes. This proportion appears realistic when comparing it to values found in similar studies (24 % for *Salmonella enterica* [9] and 51 % for *Helicobacter pylori* [10]), and considering that the large *B. cenocepacia* genome consists of a high number of non-essential genes [11] that are not all transcribed in the one growth condition analysed in the present study. Most pTSS were located on the large replicon (Table 1), which is to be expected since the large replicon harbours most essential genes [11]. Intergenic pTSS for three genes (BCAL3153, BCAL0301 and BCAL0672) were confirmed by 5′RACE (Additional file 4: Figure S1, panels A, B and C).

105 pTSS were located internal to an upstream gene (Table 1), in some cases the upstream gene was part of the same operon (Fig. 1). Two internal pTSS located in an upstream same sense gene, one with and one without LE, were analysed by 5′RACE (Additional file 4: Figure S1, panels E and F). Where a distinct LE was present, the pTSS was unambiguously confirmed, indicating that these adjacent genes do not constitute an operon. Where a LE was not apparent, transcription initiation as well as read-through from further upstream in the operon occurred. This confirms previous observations that transcription can be initiated or modulated at several loci within an operon, resulting in full length transcripts and alternative transcripts [12].

### Promoters

As the present dataset is derived from analysis of only one condition, biofilm growth, promoter search focussed on the core promoter region with its −10 and −35 elements.

**Fig. 2:**
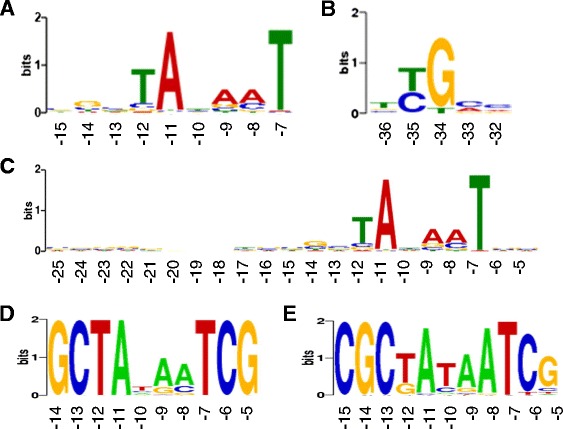
Sequence logos showing conserved motifs upstream of pTSS. Motifs are based on 1733 sequences upstream of primary TSS located in intergenic regions, analysed with Improbizer [13] (panels **a** and **b**), MEME [15] (panel **c**) and DMINDA [16] panel (**d** and **e**). X-axis: Average position relative to TSS. Y-axis: Sequence conservation

Sequences 60 nt upstream of pTSS, excluding TSS located in genomic islands, were submitted to Improbizer [13], a motif finding algorithm that considers location of sequence patterns within the input sequences and favours motifs that occur at the same place. Improbizer found 3 motifs (Additional file 5: Table S4), the first two of which were plausible candidates for a −10 and −35 box based on their sequence and their position relative to TSS. Of the 1733 analysed upstream sequences, more than 95 % possessed a 9 nt long AT-rich motif, on average at position −8 to −16 relative to the TSS (Fig. 2a, Additional file 5: Table S4). The more conserved part of this motif, with consensus sequence TAnAAT, is very similar to the conserved −10 hexamer of *E. coli* with consensus sequence TATAAT, regarding sequence and position relative to TSS [14]. A second, less conserved motif was found in 93 % of submitted sequences, it centred at position −34 (Fig. 2b). Its consensus sequence is TTGCC, making it similar to the conserved −35 box of *E. coli* [14] with consensus sequence TTGACA.

The same 1733 upstream sequences were also analysed with MEME [15], and DMINDA [16], confirming the more conserved first motif, with the same conserved part of the core promoter region containing the −10 consensus sequence TAnAAT and the same positioning relative to TSS (Fig. 2c, d, e). The second less conserved motif could not be confirmed by either MEME or DMINDA, presumably because it is too weak to be detected by algorithms which do not take the position of the motif into account.

The sequences up to 60 nt upstream of internal pTSS, asTSS, isTSS and oTSS were screened for occurrence of the conserved and AT-rich motif (Fig. 2a) to assess whether these TSS were derived from genuine transcription initiation or from sequencing artefacts. We used Motif Finder [13], a program which considers the location of the motif in query sequences when searching for matches. More than 95 % of internal pTSS were associated with a sequence match to the AT-rich motif, on average at the same position as the input motif (Additional file 5: Table S4). Furthermore, 94 % of asTSS, 92 % of oTSS and 81 % of isTSS were associated with a matching motif in the same position.

Overall, the occurrence and position of promoters for transcription initiation further corroborates that most TSS found by dRNA-Seq are *bona fide*. The lower incidence of promoters for isTSS indicates that isTSS can be also caused by TEX inhibition at strong secondary structures, as has been observed for *Streptomyces coelicolor*, another organism with high genomic GC content [17, 18].

MEME detected only 5 motifs with an e-value <0.001, only one of which, containing the proposed −10-box (Fig. 2c), was plausible as a promoter motif based on its conservation and convergence towards a specific position (Additional file 6: Table S5). DMINDA detected 16 motifs, only two of which, both AT-rich, were converging towards a specific position (Fig. 2d and e, Additional file 7: Table S6). Variations of motifs which could represent different sigma factor binding sites were not found in this analysis. Repeating the analysis with adjusted input paratmeters did not improve results. This is probably due to the relatively large number of input sequences (upstream sequences from all genes expressed under biofilm condition). Analysing subsets of these sequences, generated based on similar expression patterns in a microarray dataset [19] or on related functions, also did not result in plausible specific and conserved sigma factor binding site motifs (data not shown). A reason for this might be the large number of sigma factors encoded in the *B. cenocepacia* J2315 genome [5]. This bacterium possesses 20 sigma factors the target genes of which have not yet been characterised and which probably have overlapping target gene populations. A more in depth analysis of promoter sequences might therefore require experimental evidence regarding sigma factor target genes, generated e.g. by ChIP sequencing.

### Length of 5′ UTRs and leaderless transcripts

**Fig. 3:**
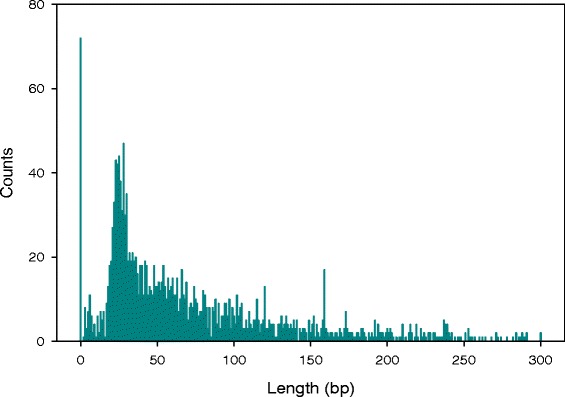
5′UTR length distribution. Data represents 5′UTRs from primary TSS located in intergenic regions, excluding internal primary TSS

The average length of 5′UTRs is 72 nt (Fig. 3), with a distribution peak between 21 and 30 nt; 75 % of 5′UTRs were between 17 and 126 nt long. This is in good agreement with values for other bacteria such as *Salmonella enterica* [9], *Helicobacter pylori* [10] and *Streptomyces coelicolor* [18].

The length of a 5′UTR can be related to expression regulation of the corresponding gene. Long 5′UTRs may contain riboswitches or provide binding sites for small regulatory RNAs [20, 21]. Leaderless genes are translated by a different mechanism than genes with a leader sequence, and have been shown to be differentially regulated under stress conditions compared to leader-lead genes [22]. To investigate a possible link between length of 5′UTRs and gene function in *B. cenocepacia* J2315, we performed functional enrichment analysis on sub-sets of genes, genes without (≤10 nt) 5′UTR or with a long (>150 nt) 5′UTR.

The pTSS of 72 genes were located exactly at the annotated start codon and the pTSS of a further 42 genes was located ≤10 nt upstream of the annotated start codons. These transcripts were considered to be leaderless. 24 leaderless transcripts were tRNAs, consistent with the length of tRNA leader sequences in *B. cenocepacia* J2315, which ranges from 5 to 127 nt (see below). Functional enrichment analysis of the remaining leaderless transcripts revealed that, with 28 coding sequences (CDS), transcriptional regulators of various families are particularly over-represented (Additional file 8: Table S7). The TA-rich promoter motif was found directly upstream of genes with leaderless transcripts (Fig. 3), like reported for other bacteria [23], showing that leaderless genes possess a transcription initiation signal instead of a Shine-Dalgarno sequence.

187 CDS featured a long 5′UTR of >150 nt. Functional enrichment analysis performed on these genes revealed transcriptional regulators, nucleotide binding and membrane proteins as over-represented (Additional file 8: Table S7). Comparison of the respective sequences with the Rfam database revealed the yet unannotated S-adenosyl-L-homocysteine (SAH) riboswitch [20] in the 5′UTR of an adenosylhomocysteinase (BCAL0145, see below).

### Transcription initiation in genomic islands

**Fig. 4:**
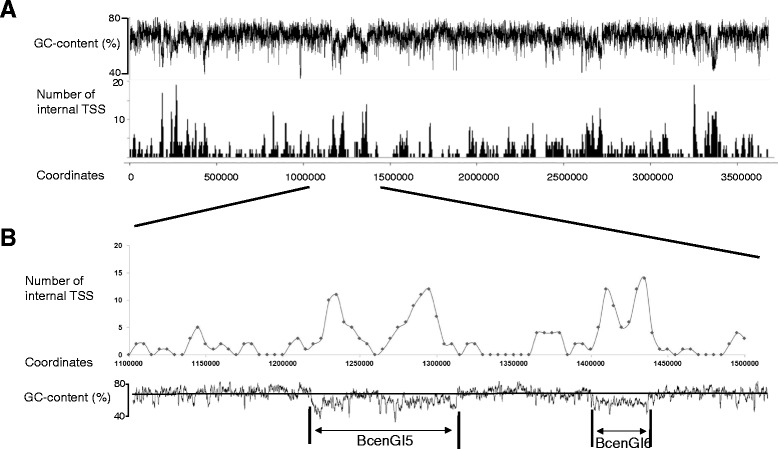
Distribution of internal TSS across replicon 1 of the *B. cenocepacia* J2315 genome. Data represents numbers of isTSS and asTSS within 10000 bp sliding windows and 5000 bp steps. Panel **a**: Number of internal TSS across the whole replicon 1. Panel **b**: Detail for genome positions 1,100,000–1,500,000 of replicon 1, including two genomic islands with a GC-content lower than the genome average. Position of genomic islands: BcenGI5 1222566..1315385, BcenGI6 1402882..1437088. The window size for displaying the GC-content is 500, the horizontal line depicts the genome average of 66.9 %

The genome of *B. cenocepacia* J2315 contains 14 genomic islands with a GC-content lower than genome average of 66.9 % or with CDS similar to prophages; these GI encompass 9.3 % of the total genome [5]. Internal TSS appear to occur at a higher density in genomic islands (Fig. 4), in agreement with observations made in *E. coli* and *Salmonella* sp. [24]. 18 % of all annotated isTSS and 25 % of asTSS are located in genomic islands, which is higher than expected given the proportion of genomic islands on the genome.

This indicates that, while genes in genomic islands can be transcribed, these loci are not completely compatible with the *B. cenocepacia* J2315 transcriptional machinery, and transcription initiation often does not result in a functional product [25].

Interestingly, the genomic region encoding exopolysaccharide synthesis genes implicated in capsule formation (BCAL3217-3246) also shows a higher density of internal TSS, thus confirming that this region was acquired by recent gene transfer and constitutes a genomic island [26]. Moreover, most TSS annotated on the plasmid were categorised as internal TSS, indicating that plasmid genes are also not entirely compatible with *B. cenocepacia* transcription mechanisms. We propose that the distribution of internal TSS across the *B. cenocepacia* genome can be used as a further criterion to identify genomic islands in *B. cenocepacia*, as genomic islands can display a higher density of internal TSS than genome background.

### Re-annotation of start codons and discovery of un-annotated proteins

Gene calling for genome annotation usually predicts up to 99 % of all protein coding genes correctly, but the rate of gene calling errors can rise to 14 % in the GC-rich *Burkholderia* sp. genomes [27]. We used TSS mapping to screen for mis-annotated and un-annotated genes in the *B. cenocepacia* 2315 genome.

For 72 genes, 66 CDS and 6 RNAs, TSS mapping predicted the primary TSS internal and downstream of the annotated gene start, suggesting that an incorrect start codon might have been predicted and the gene is shorter than annotated. The internal TSS position for BCAL0063 was confirmed by 5′RACE (Additional file 4: Figure S1, panel D) as an example. All genes with internal and downstream pTSS, as well as leaderless genes, were screened for alternative start codons downstream of their annotated gene start. Unusually long 5′UTRs on the other hand could indicate that the corresponding gene is longer than annotated. 5′UTRs longer than 150 nt, as well as orphan TSS consistent with a 5′UTR according to gRNA-Seq data, were screened for open reading frames and upstream alternative start codons. 64 CDS for which an alternative start codon could be predicted, making the gene either longer or shorter, are listed in Table 2.

**Table 2:** Alternative start codons for CDS with internal TSS or long 5′ UTRs ≥ 150 nt, as predicted by Prodigal

TSS position	Strand	Gene	Annotated gene position	Alternative gene start	Annotated start codon	Alternative start codon	Alternative gene is
Replicon 1							
108753	+	BCAL0088	108758..109138	108842	GTG	ATG	shorter
175808	+	BCAL0151	175965..177107	175872	ATG	ATG	longer
196936	+	BCAL0175	196857..197147	196965	ATG	ATG	shorter
317647	+	BCAL0289	317651..322474	317771	GTG	ATG	shorter
566857	-	BCAL0515	Complement (565664..566551)	566617	ATG	ATG	longer
700318	+	BCAL0646	700302..701309	700341	GTG	ATG	shorter
787134	+	BCAL0722	787317..788582	787293	GTG	GTG	longer
937522	+	BCAL0865	937677..938504	937566	TTG	ATG	longer
999283	-	BCAL0916	Complement (998559..999290)	999245	ATG	ATG	shorter
1041114	-	BCAL0952	Complement (1040227..1040955)	1041075	ATG	ATG	longer
1147555	+	BCAL1059	1147734..1148927	1147695	ATG	ATG	longer
1161150	-	BCAL1069	Complement (1159161..1160903)	1160996	ATG	ATG	longer
1207883	-	BCAL1102	Complement (1207594..1207914)	1207848	GTG	ATG	shorter
1390938	-	BCAL1277	Complement (1388805..1390997)	1390868	ATG	ATG	shorter
1463359	-	BCAL1335	Complement (1462753 ..1463376)	1463334	ATG	ATG	shorter
1892553	+	BCAL1715	1892545..1893150	1892578	GTG	GTG	shorter
1933621	+	BCAL1753	1933609..1934565	1933672	ATG	ATG	shorter
2041280	-	BCAL1849	Complement (2040670..2041326)	2041221	ATG	ATG	shorter
2067405	-	BCAL1871	Complement (2065350..2067203)	2067380	ATG	ATG	longer
2120545	+	BCAL1921	2120767..2121222	2120662	GTG	ATG	longer
2136329	+	BCAL1937	2136797..2138446	2136644	ATG	ATG	longer
2656999	-	BCAL2401	Complement (2656576..2657001)	2656953	GTG	ATG	shorter
2822661	-	BCAL2559	Complement (2822212..2822745)	2822661	TTG	ATG	shorter
3011653	+	BCAL2740	3011646..3012689	3011679	ATG	ATG	shorter
3037852	-	BCAL2766	Complement (3037324..3037656)	3037734	TTG	ATG	longer
3093624	-	BCAL2818	Complement (3092132..3093673)	3093598	GTG	ATG	shorter
3094006	+	BCAL2819	3093965..3095413	3094037	GTG	ATG	shorter
3122838	-	BCAL2841	Complement (3121514..3122860)	3122710	GTG	ATG	shorter
3257864	-	BCAL2974	Complement (3257551..3257931)	3257820	ATG	ATG	shorter
3266225	+	BCAL2981	3266173..3268080	3266245	ATG	ATG	shorter
3461898	+	BCAL3168	3461889..3462575	3461937	ATG	ATG	shorter
3533713	-	BCAL3229	Complement (3531274..3533205)	3533499	GTG	ATG	longer
3584399	-	BCAL3275	Complement (3583374..3584396)		ATG	TTG	shorter
3619212	-	BCAL3302	Complement (3616817..3619048)	3619129	GTG	ATG	longer
3666930	+	BCAL3349	3666923..3667387	3666965	ATG	ATG	shorter
3721637	-	BCAL3395	Complement (3719319..3721676)	3721619	ATG	ATG	shorter
Replicon 2							
14979	+	BCAM0014	15152..15757	15110	ATG	ATG	longer
184425	-	BCAM0158	Complement (182630..184141)	184234	ATG	ATG	longer
570490	+	BCAM0516	570437..570919	570545	TTG	ATG	shorter
713692	+	BCAM0645	713658..715211	713733	ATG	ATG	shorter
878428	+	BCAM0795	878392..878856	878428	TTG	ATG	shorter
904562	+	BCAM0820	904770..905861	904728	ATG	ATG	longer
1009355	+	BCAM0918	1009947..1011812	1009395	GTG	ATG	longer
1203868	+	BCAM1112	1204104..1206368	1204068	ATG	ATG	longer
1405396	+	BCAM1280	1405387..1406880	1405435	ATG	ATG	shorter
1419414	+	BCAM1290	1419396..1420382	1419438	TTG	ATG	shorter
1967577	+	BCAM1756	1967535..1969922	1967598	GTG	ATG	shorter
2032438	+	BCAM1814	2032438..2034045	2032462	ATG	ATG	shorter
2291422	-	BCAM2058	Complement (2290470..2291126)	2291303	TTG	ATG	longer
2291441	+	BCAM2059	2291632..2292495	2291587	ATG	ATG	longer
2300468	+	BCAM2066	2300462..2301877	2300489	ATG	ATG	shorter
2471730	-	BCAM2210	Complement (2471467..2471733)	2471658	ATG	ATG	shorter
2613643	+	BCAM2327	2613607..2614722	2613697	ATG	ATG	shorter
2703044	+	BCAM2401	2703049..2703798	2703109	ATG	ATG	shorter
2703867	+	BCAM2402	2703876..2704169	2703900	GTG	ATG	shorter
3026129	+	BCAM2679	3026111..3026374	3026153	TTG	ATG	shorter
3058373	-	BCAM2703	Complement (3057460..3058437)	3058353	GTG	ATG	shorter
3077665	-	BCAM2719	Complement (3077106..3077669)	3077606	ATG	ATG	shorter
3133147	+	BCAM2769	3133305..3134042	3133281	GTG	ATG	longer
Replicon 3							
69880	-	BCAS0060	Complement (68555..69688)	69709	ATG	ATG	longer
80842	-	BCAS0070	Complement (79501..80850)	80742	TTG	ATG	shorter
769919	+	BCAS0706	770234..771562	770120	TTG	ATG	longer
Plasmid							
2363	+	pBCA003	2363..3220	2546	ATG	ATG	shorter
79981	-	pBCA080	Complement (79541..79750)	79891	ATG	ATG	longer

**Fig. 5:**
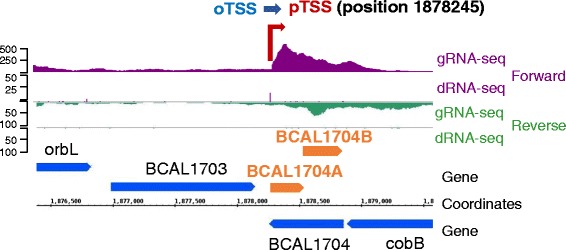
Re-annotation of a conserved hypothetical protein as a type II toxin-antitoxin system. New annotation: BCAL1704A, ParD, antitoxin component of, and BCAL1704B, ParE, toxin component of a type II toxin-antitoxin system. Position of ParD: 1878263..1878505. Position of ParE: 1878498..1878794. The start of ParE overlaps the 3′end of ParD by 8 nt. dRNA-Seq data are represented as read starts per base, gRNA-Seq data are represented as coverage

To search the genome for un-annotated protein-coding genes, all oTSS and asTSS possessing a LE were screened for an open reading frame with an ATG start codon which could produce a protein ≥50 amino acid residues. These amino acid sequences were compared to the NCBI protein sequence database and hits with both >75 % query coverage and >40 % amino acid identity were retained (Additional file 9: Table S8). Most hits were annotated as hypothetical or conserved hypothetical proteins with no predicted function. In one case, a type II toxin-antitoxin module was discovered on the opposite strand of a gene currently annotated as BCAL1704, a conserved hypothetical protein. We propose to re-annotate this loci, to BCAL1704A, a ParD-type antitoxin with 81 amino acid residues, and BCAL1704B, a ParE-type toxin with 99 amino acid residues (Fig. 5).

### Antisense transcription

Most reads mapping to annotated genome features map in sense direction (>93 %), only 5–7 % map antisense, based on the gRNA-Seq dataset. Nonetheless, antisense transcription is pervasive in *B. cenocepacia* J2315, and reads map antisense to nearly all genome features (Additional file 10: Table S9). However, it is safe to assume that not all of these antisense transcripts have a function [28]. Genes with strong sense transcription tend to have a high number of antisense reads, likely a result of technical RNA sequencing artefacts. Moreover, antisense transcription can be a result of read-through from the 3′ end of a downstream opposite-sense gene or transcription initiation for an upstream opposite-sense gene.

We attempted to annotate antisense transcripts that might genuinely be involved in gene regulation, i.e. transcripts complementary to at least part of a gene and not belonging to any category mentioned above. For this purpose we filtered for genes with a minimum antisense-RPKM of 10 and a ratio of antisense-to-sense RPKM of >0.1, leaving 11 % of all genes. 105 of these featured an asTSS, or an oTSS located ≤ 300 bp downstream of the 3′end of a gene, with a LE in gRNA-Seq data (Additional file 11: Table S10). Among the genes with antisense transcription were 12 transposases, 12 transcriptional regulators and two toxin-antitoxin systems, the same categories for which functional antisense transcripts have been found in other bacteria [29]. These antisense RNAs might therefore also have a function in *B. cenocepacia*.

### Genes induced under biofilm condition

To screen the RNA-seq data for genes potentially essential for biofilm growth of *B. cenocepacia* J2315, we compared RNA-seq data with published microarray datasets obtained from cells grown in a biofilm [30], from planktonic cells harvested in stationary phase, and from cells grown under reduced oxygen levels [19] as well as under various stress conditions [19, 30]. The aim was to find genes with high expression in biofilms (gRNA-Seq RPKM >100) and induced in biofilms while not induced under any other condition.

**Fig. 6:**
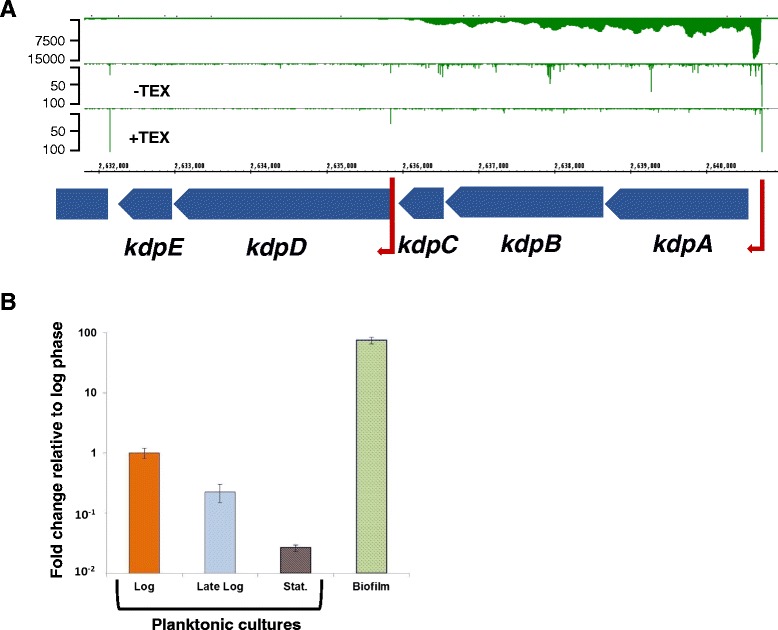
Expression of the *kdp*-system in *B. cenocepacia* J2315 biofilms. Panel **a**: dRNA-Seq and gRNA-Seq data for the kdp-operon. dRNA-Seq data are represented as read starts per base, gRNA-Seq data are represented as coverage. Red arrows: pTSS, at position 2640727 (*kdpA*) and 2635837 (*kdpD*). Panel **b**: Quantitative RT-PCR of *kdpA* in biofilms and planktonic cultures. Bars represent expression relative to planktonic logarithmic cultures and are based on three biological replicates. Orange: Planktonic culture in logarithmic phase, harvested at 5 × 10^8^ CFU/ml. Light blue: Planktonic culture in late logarithmic phase, harvested at 1 × 10^9^ CFU/ml. Brown: Planktonic culture in stationary phase, harvested at 2.2 × 10^9^ CFU/ml. Green: Biofilm grown in microtiter plates

Only the first two genes of a multi-subunit K^+^-transport system (*kdpA*-*kdpE*, BCAL2379-2383) met these criteria. These five genes are organised in two operons with two annotated TSS, the first of which is 100-fold stronger expressed than the second (Fig. 6a). The first operon contains the structural K^+^-transport and ATPase genes, the second contains the two-component regulatory system required for induction of *kdpABC*. qPCR analysis of *kdpA* confirmed its induction in biofilms compared to planktonic cultures in logarithmic and stationary phase (Fig. 6b). In *E. coli*, the Kdp-system is an inducible high affinity K^+^-pump essential for intracellular K^+^-homoeostasis under salt stress, [31]. The two-component system *kdpDE* has also been implicated in virulence in various pathogenic bacteria [31]. In *Bacillus* sp., the Kdp-system was found to be up-regulated in swarming cells [32] and necessary for biofilm formation [33], and the Kdp-system was up-regulated in *Staphylococcus aureus* biofilms [34]. These observations indicate that, apart from its role in osmoadaptation, the Kdp-system also plays an important role in biofilm formation, presumably also in *B. cenocepacia*. Work to characterise the relevance of these genes for *B. cenocepacia* biofilm formation and persistence, using deletion and conditional mutants, is ongoing.

### Transcription of annotated non-coding RNAs

The published genome annotation [5] includes rRNAs and small non-coding RNAs such as tRNAs, several riboswitches and essential RNAs.

For 47 of the 74 annotated tRNA genes a TSS was found, positioned 5–127 nucleotides upstream of the annotated gene start. Most tRNA transcripts are, as expected, processed at position +1 of the annotated gene, marked by a local maximum in read starts in the -TEX library and depletion in the + TEX library.

Exceptions are the tRNAs for histidine and selenocysteine, which are processed at position −1 and −10, respectively. 16S rRNA genes have a TSS at position −237, the co-transcribed 23S and 5S rRNA are processed at +3 and +2, respectively. TSS for annotated riboswitches are positioned at annotated gene start (thiamine pyrophosphate) and 5 nt (glycine) or 8–14 nt upstream (cobalamin).

Of the annotated essential RNAs, transfer messenger RNA has an 11 bp 5′-leader element. RNase P appears longer than annotated, its TSS is located at position −51 and the transcript shows no obvious processing site. The signal recognition particle appears shorter than annotated, with a TSS at position +2.

Overall, dRNA-Seq data are in good agreement with annotated non-coding RNAs, showing that small RNAs are detected with our experimental approach.

### Candidate regulatory small RNAs

Apart from annotated tRNAs, riboswitches and conserved essential RNAs, bacterial genomes also contain other small non-coding RNAs that are involved in post transcriptional gene expression regulation [21, 35]. For a preliminary screening of TSS for highly expressed novel small RNAs, sequences following oTSS, isTSS and asTSS, as well as 5′UTRs longer than the average of 72 nt, were compared to the Rfam database.

In this manner nine small RNAs were found in the *B. cenocepacia* J2315 genome (Table 3). One of these constitutes the 6S RNA, which was already predicted and its expression confirmed by other studies on *B. cenocepacia* [30, 36]. Two small RNAs are phage-related regulatory RNAs located on genomic island BcenGI9. Two Rfam hits constitute conserved regulatory motifs: the SAH riboswitch located in the 5′UTRs of BCAL0145, an adenosylhomocysteinase, and the *Burkholderiales*-specific sucA RNA motif located in the 5′UTR of BCAL1515, *sucA*, an enzyme of the citric acid cycle. The sucA RNA motif probably constitutes a riboswitch [37]. The remaining four novel small RNAs all are from the same family, “toxic small RNAs”, which were found to be toxic if introduced into *E. coli* on a cloning vector [38]. Expression of these toxic small RNAs has been confirmed by Northern blotting in four strains of *B. cenocepacia*, including strain J2315 [38]. However, their function in *B. cenocepacia* is unknown.

**Table 3:** Novel non-coding small RNAs in *B. cenocepacia* J2315 with hits in Rfam database

Preliminary name	Strand	Length (nt)^b^	Terminator sequence	Adjacent genes		Relative orientation	Genome position	Rfam ID	Name
ncS03	+	58	yes	BCAL0197	BCAL0198	→ → ←	221314..221371	RF02278	Toxic small RNA
ncS05	-	67	yes	BCAL0436	BCAL0437	← ← →	Complement (479440..479506)	RF02278	Toxic small RNA
ncS17	+	200	yes	BCAL2667	BCAL2668	→ → →	2935785..2935984	RF00013	6S RNA
ncS23	+	81	yes	BCAL2965	BCAL2965a	→ → →	3246834..3246914	RF01394	isrK
ncS24	+	90	no	BCAL2965	BCAL2965a	→ → →	3246937..3247026	RF01695	C4^a^
ncS27	-	92	yes	BCAL3348a	BCAL3349	→ ← →	Complement (3666557..3666648)	RF02278	Toxic small RNA
ncS62	+	57	yes	BCAM1871	BCAM1872	→ → ←	2089713..2089769	RF02278	Toxic small RNA
ncR1	+	119	no	BCAL0144	BCAL0145	→ → →	168973..169091	RF01057	SAH riboswitch
ncR2	+	118	no	BCAL1514	BCAL1515	→ → →	1676458..1676575	RF01070	SucA RNA motif

^a^C4 forms one transcriptional unit with isrK

^b^Length of non-coding RNAs is inferred from dRNA-Seq data and terminator structures (when present) and is not yet experimentally confirmed

The sequence of small RNAs is generally only conserved between closely related bacterial species and can vary dramatically in primary sequence and secondary structure between bacterial genera [39]. On the other hand, most small RNAs in the Rfam database are derived from well-studied species such as *E. coli*, which are not closely related to *Burkholderia*. Since the Rfam algorithm first performs a BLAST search, functional homologues from distantly related species are unlikely to produce a hit and novel small RNAs specific for *B. cenocepacia* are likely to be overlooked by this approach.

To identify putative regulatory small non-coding RNAs not yet represented in the Rfam database, we compared sequences derived from oTSS to non-coding RNAs experimentally confirmed in *B. cenocepacia* strains in other studies using RNA-sequencing and Northern blotting [36], co-purification with Hfq-protein [40] or microarrays [30, 41–43], if they showed the following properties: strong transcription initiation with a coverage >300 reads in dRNA-Seq data, a defined 3′ end in dRNA-Seq data or a transcript appearing short (<500 nt), truncated or missing in gRNA-Seq data.

Homologues of six short transcripts with strong transcription initiation from oTSS were also expressed in *B. cenocepacia* strains AU1054 and HI2424 under conditions mimicking the human lung and the soil environment (Table 4) [36]. One of these was confirmed to be a small RNA by Northern blotting in the same study. One short transcript was present in the RNA fraction co-purified with the Hfq-protein of *B. cenocepacia* J2315 [40]. Hfq is an RNA chaperone which mediates base pairing of small regulatory RNAs with their target mRNA [44] and *B. cenocepacia* J2315 harbours the Hfq gene as two non-identical homologues [5], making this non-coding RNA a plausible candidate for a regulatory small RNA. These findings show that with the approach used in this study we could identify transcripts which could encode non-coding regulatory small RNAs. Their strong expression in biofilms suggests that these small RNAs might have a role in adaptation of *B. cenocepacia* to biofilm conditions.

**Table 4:** Putative novel non-coding RNAs expressed in *B. cenocepacia* and experimentally confirmed by previous studies

Preliminary name	Strand	Length (nt)^b^	Terminator sequence	Adjacent genes		Relative orientation	Genome position	Replicon	Type of experiment
ncS04	+	105	yes	BCAL0264	BCAL0265	← → ←	292949..293053	1	Co-purification with Hfq [40]
ncS06	+	117	no	BCAL0549	BCAL0550	→ → ←	603652..603828	1	RNA-Seq and Northern blot [36]
ncS11	-	208	yes	BCAL2293	BCAL2294	→ ← ←	Complement (2545296..2545503)	1	RNA-Seq [36]
ncS18	+	178	no	BCAL2713	BCAL2714	→ → →	2979006..2979183	1	RNA-Seq [36]
ncS21^a^	+	361	yes	BCAL2737	BCAL2738	→ → →	3008232..3008591	1	RNA-Seq [36]
ncS33	+	93	no	BCAM1725	BCAM1726	→ → →	1926664..1926756	2	RNA-Seq [36]
ncS36	+	60	no	BCAM2207	BCAM2208	← → →	2468880..2468939	2	RNA-Seq [36]

^a^ncS21 is associated with an open reading frame and potentially constitutes a protein, see Additional file 6: Table S5

^b^Length of non-coding RNAs is inferred from dRNA-Seq data and terminator structures (when present) and is not yet experimentally confirmed

A detailed analysis of putative novel small non-coding RNAs expressed in *B. cenocepacia* J2315 biofilms and their involvement in biofilm formation is ongoing.

## Conclusions

This study is the first genome-wide analysis of TSS in *B. cenocepacia*. Through differential RNA-Sequencing, bioinformatics methods and 5′RACE we annotated the primary TSS for 2089 genes expressed in biofilms, defined alternative start codons for 64 genes, identified novel protein sequences and characterised antisense transcription. 15 non-coding RNAs highly expressed in biofilms and a potassium uptake system strongly induced under biofilm conditions were identified that could be involved in biofilm formation and survival. Comparison of dRNA-Seq data with gRNA-Seq data proved to be invaluable for TSS categorisation and interpretation.

The data presented in this study will provide the starting point for evaluation of the regulatory processes involved in *B. cenocepacia* biofilm formation and could reveal novel targets for antibiotic therapy.

## Methods

### Bacterial strain and culture conditions

*B. cenocepacia* strain J2315 (LMG 16656) was grown in Luria-Bertani broth (LBB, Oxoid, Hampshire, UK) at 37 °C. Biofilms were grown in 96-well microtiter plates and cells were harvested as described previously [45]. Planktonic cultures were grown in 250 ml glass flasks, incubated at 37 °C in a shaking incubator at 150 rpm and harvested as described previously [46].

### RNA extraction and sequencing

For differential RNA-sequencing (dRNA-Seq, [6]), total RNA was extracted from cell pellets of two biological biofilm replicates using the RiboPure Bacteria kit (Life Technologies, Renfrewshire, UK). RNA samples were then split and one aliquot treated with Terminator™ 5′ monophosphate-dependent exonuclease (TEX). A separate library was constructed from each aliquot, TEX-treated and untreated. rRNA depletion and RNA fragmentation steps were omitted. 5 'triphosphates were removed using tobacco acid pyrophosphatase and an RNA adapter was ligated to the 5′-monophosphate of the RNA. RNA was then polyadenylated and first-strand cDNA synthesis performed using an oligo(dT)-adapter primer. The resulting cDNA was PCR-amplified to about 20-30 ng/μl. A library-specific barcode for multiplex sequencing was part of a 3′-sequencing adapter. The following adapter sequences flank the cDNA inserts: TrueSeq Sense primer 5′AATGATAC GGCGACCACCGAGATCTACACTCTTTCCCTACACGACGCTCTTCCGATCT-3′ and TrueSeq Antisense NNNNNN primer (NNNNNN = 6n barcode for multiplexing) 5′-CAAGCAGAAGACGGCATACGAGAT-NNNNNN-GTGACTGGAGTTCAGACGTGTGCTCTTCCGATC(dT25)-3′. The resulting cDNA libraries were sequenced using a HiSeq 2000 machine (Illumina) in single-read mode and running 100 cycles.

For global transcriptome sequencing (gRNA-Seq), RNA of three biological biofilm replicates was extracted as described previously [45]. Total RNA was depleted of ribosomal RNA using the Ribo-zero Magnetic Gram-Negative Bacteria kit (Epicentre, Madison, WI, USA) and used for Illumina paired-end sequencing generating 100 bp reads [45].

### Mapping of RNA seq reads

In order to assure a high sequence quality, the Illumina reads in FASTQ format were trimmed with a cut-off phred score of 20 by the program fastq_quality_trimmer from FASTX toolkit version 0.0.13 (http://hannonlab.cshl.edu/fastx_toolkit/). The following steps were performed using the subcommand “create”, “align” and “coverage” of the tool READemption [47] version 0.3.5. The poly(A)-tail sequences were removed and a size filtering step was applied in which sequences shorter than 12 nt were eliminated. The collections of remaining reads were mapped to the reference genome sequences (accessions AM747720, AM747721, AM747722, and AM747723) using segemehl software version 0.2.0 [48]. Coverage plots in wiggle format representing the number of aligned reads per nucleotide were generated based on the aligned reads just considering the first base of each read.

### TSS annotation and classification

Mapping output from dRNA-Seq was split by replicon and coverted to SAM format (using samtools 1.2). Those SAM files were used as input for TSSAR, a tool for automated de novo TSS annotation [49], to map all loci with coverage maxima which are enriched in the TEX-treated library. Default parameters (p-value threshold 0.001, noise threshold 2, merge range 5) were used, the two biological replicates were pooled and TSS within 5 nt of each other were clustered into one. Genome regions with read start distributions that do not conform to a Poisson distribution are omitted from TSSAR analysis [49]. Such regions were then manually annotated by scanning the respective wiggle files for nucleotides with an abrupt increase in coverage; except regions internal to rRNA genes where the abundance of mapped reads was too high to allow robust TSS annotation. A noise filter with a minimum coverage of 10 read starts was applied, based on the normalised number of read starts per base, to ensure reported TSS are robust. TSS were then classified according to their genomic context. All TSS positions were assigned relative to the start of the associated annotated gene, with the first base of the gene being position +1, upstream positions start with −1.

We used the *B. cenocepacia* strain J2315 annotation deposited in the EMBL database under accession numbers AM747720, AM747721, AM747722, and AM747723 [5] for assigning and classifying TSS. This newer annotation includes 21 non-coding RNAs other than rRNAs and tRNAs: riboswitches for e.g. thiamine and cobalamin and the essential RNAs tmRNA, ribonuclease P and bacterial signal recognition particle. In contrast to this, the older annotation deposited at NCBI under accession numbers NC_011000, NC_011001, NC_011002 and NC_011003 contains only rRNAs and tRNAs and lacks all genes annotated as pseudogenes in the newer annotation.

### Rapid amplification of cDNA ends (RACE)

To confirm TSS, the 5′end of selected transcripts was determined by RACE. For 5′RACE, RNA was transcribed with gene specific primers and a homopolymeric tail added to the 3′ end of the resulting cDNA. The tailed cDNA was then amplified with nested gene specific primers and a primer complementary to the homopolymer tail. We used the 5′ RACE System for Rapid Amplification of cDNA Ends (Life Technologies, Paisley, UK) with the following changes to standard protocol: the reverse transcriptase provided with the kit was replaced by ThermoScript reverse transcriptase (Life Technologies, Paisley, UK), temperature for first strand synthesis was elevated to 60 °C and the additional protocol for transcripts with high GC-content was followed. The resulting amplicons were cloned into *E. coli* using the pGEM®-T Vector system (Promega, WI, USA) and JM109 high-efficiency competent cells (Promega, WI, USA). Vector inserts amplified from clones were analysed by Sanger-sequencing. RACE primer sequences are available as supplementary data (Additional file 12: Table S11).

### Quantitative RT-PCR analysis

For quantitative RT-PCR (qPCR), planktonic and biofilm cultures were grown and harvested as described previously [45, 46]. RNA extraction from cell pellets was performed with a modified protocol, using the RiboPure Bacteria kit (Life Technologies, Renfrewshire, UK) with the following changes to manufacturer’s instructions: before transferring the RNA to the filter cartridge, 1.25 instead of 0.5 volumes of ethanol were added to retain a higher proportion of small RNAs. Before DNase treatment, RNA was denatured by heating to 65 °C for 5 min and DNase incubation time was increased from 30 min to 60 min. The RNA extract was then DNase-digested (NEB, Ipswich, MA, USA) for a second time for 60 min and extracted with phenol-chloroform (Roti-Aqua-P/C/I for RNA extraction, Carl Roth, Karlsruhe, Germany). Extracted RNA was precipitated with 2.5 volumes ethanol-sodium acetate mix (ethanol : 3 M Na-acetate 30:1, pH 6.5) over night at −20 °C, centrifuged and washed with 70 % ethanol. The RNA pellet was air dried and re-dissolved in water.

cDNA generation and quantitative RT-PCR was performed as described previously [50] using eight control genes with minimal expression changes across all tested conditions for data normalisation. All eight control genes were used for normalisation in every condition. Primer sequences of target and control genes are shown in Additional file 10: Table S9.

### Further bioinformatical analysis

dRNA-Seq and gRNA-Seq data were visualised with the Integrated Genome Browser version 8.3.1. [51] for manual comparison. Novel proteins were searched for by comparing sequences to the NCBI non-redundant protein sequence database using pBLAST [52], novel non-coding small RNAs were searched for by comparing DNA sequences to the Rfam database [39]. The TransTerm algorithm [53] was used to screen for Rho-independent transcriptional terminator structures. Functional enrichment analysis was performed with the DAVID web tool [54], using a custom background gene list consisting of all genes with an assigned pTSS. Alternative start codons were predicted using Prodigal [55]. DNA sequence motifs upstream of pTSS were identified with Improbizer [13] using default parameters and motifs were then searched for in sequences upstream TSS belonging to other categories with Motif Matcher [13]; both programs consider location of the motif. Improbizer and Motif Matcher are available as web tools at https://users.soe.ucsc.edu/~kent/improbizer/index.html. Sequences upstream of pTSS were also submitted to MEME [15] and DMINDA [16] for comparison and cross-validation. Input parameters were default, except for minmum and maximum motif length in MEME which were 8 and 50, respectively.

### Supporting data and software

The dRNA-Seq raw sequencing data was submitted to ArrayExpress under accession number E-MTAB-3381. The gRNA-Seq raw reads are available in ArrayExpress under accession number E-MTAB-2079 [45]. A script that performs the READemption and TSSAR based analysis can be retrieved from https://zenodo.org/record/17358 (DOI: 10.5281/zenodo.17358).

Plots for read starts per nucleotide from dRNA-Seq and for coverage from gRNA-Seq as well as data for TSS, candidate regulatory RNAs and alternative annotations are available on the *Burkholderia* genome database [56] beta site (www.burkholderia.com).

## Additional files

Additional file 1: Table S1. Number of mapped reads per RNA sample. (XLSX 11 kb) Additional file 2: Table S2. Transcription start sites identified by differential RNA sequencing and automated annotation. (XLSX 489 kb) Additional file 3: Table S3. Annotated and categorised TSS in the *B. cenocepacia* J2315 genome. (XLSX 353 kb) Additional file 4: Figure S1. Transcription start sites confirmed by 5′RACE. dRNA-Seq data are represented as number of read starts per base and gRNA-Seq data as read coverage, both are visualised using the Integrated Genome Browser [51]. dRNA-Seq data are represented as read starts per base, gRNA-Seq data are represented as coverage. Blue arrows depict representative sequences derived from 5′RACE analysis. Panel A: pTSS of BCAL3153, confirmed by 2 out of 8 RACE sequences, 6 sequences were shorter than the putative 5′UTR. Panel B: pTSS of BCAL0672, confirmed by 5 out of 12 RACE sequences, 7 sequences were shorter than the putative 5′UTR. The isTSS internal to BCAL0672 was not confirmed by 5′RACE, transcripts originating from this locus are probably truncated. Panel C: pTSS of BCAL0301 confirmed by 2 out of 7 RACE sequences, 5 sequences were shorter than the putative 5′UTR. The isTSS located internal to BCAL0300 could not be confirmed, transcripts originating from this locus are probably truncated. Panel D: Internal pTSS of BCAL0063. 4 out of 5 RACE sequences confirmed the internal pTSS, one read was shorter. Panel E: isTSS within BCAL3201: 3 out of 9 RACE sequences confirmed the isTSS, 3 were shorter and 3 longer than the transcript originating at this TSS. Sequences were therefore derived from transcripts originating from this TSS as well as from TSS further upstream in the operon. Panel F: pTSS for BCAL3391, internal to BCAL3390: confirmed by 6 out of 6 RACE sequences. (PDF 114 kb) Additional file 5: Table S4. Promoter motifs in sequences upstream of TSS based on Improbizer [13] analysis. The last nt of all sequences is position +1 of the transcript, the TSS. Motifs with scores >2 were regarded as positive matches. Motif sequences are indicated in capital letters. (XLSX 436 kb) Additional file 6: Table S5. Promoter motifs based on MEME [15] analysis. (XLSX 124 kb) Additional file 7: Table S6. Promoter motifs based on DMINDA [16] analysis. Motif sequences are shown in context with the expression pattern of the respective genes, based on microarray data [19]. (XLSX 319 kb) Additional file 8: Table S7. Genes belonging to enriched functionally related gene groups among leaderless transcripts and among transcripts with a 5′UTR longer than 150 nt. (XLSX 12 kb) Additional file 9: Table S8. Amino acid sequences associated with orphan TSS or internal antisense TSS and with homologues in the non-redundant protein sequence database. (XLSX 18 kb) Additional file 10: Table S9. Number of mapped reads and RPKM values sense and antisense for all genes of the *B. cenocepacia* J2315 genome. (XLSX 2108 kb) Additional file 11: Table S10. Genes with antisense transcripts. (XLSX 20 kb) Additional file 12: Table S11. Oligonucleotides used in this study. (DOCX 17 kb)

## Abbreviations

Bcc: *Burkholderia cepacia* complex
BcenGI: *Burkholderia cenocepacia* genomic island
bp: Base pairs
cDNA: Complementary DNA
CDS: Coding sequence
CFU: Colony forming units
dRNA-Seq: Differential RNA sequencing
gRNA-Seq: Global RNA sequencing
Hfq: Host factor q
kdp: Potassium transport pump
LBB: Luria-Bertani broth
LE: Leading edge of transcription
Mb: Mega base pairs
nt: Nucleotide
NTP: nucleoside triphosphate
TEX: Terminator exonuclease
tRNA: Transfer RNA
rRNA: Ribosomal RNA
RACE: Rapid amplification of cDNA ends
RPKM: Reads per kilo base per million mapped reads
SAH: S-adenosyl-L-homocysteine
UTR: Untranslated region
TSS: Transcription start site (pTSS: Primary TSS, asTSS: Internal antisense TSS, isTSS: Internal sense TSS, oTSS: Orphan TSS, sTSS: Secondary TSS)
