# Supplementary figures and images for: Genome-wide transcription start site profiling in biofilm-grown Burkholderia cenocepacia J2315
Source: BMC Genomics. 2015 Oct 13;16:775. doi: 10.1186/s12864-015-1993-3 (PMC4603805; doi:10.1186/s12864-015-1993-3)

Figure S1A

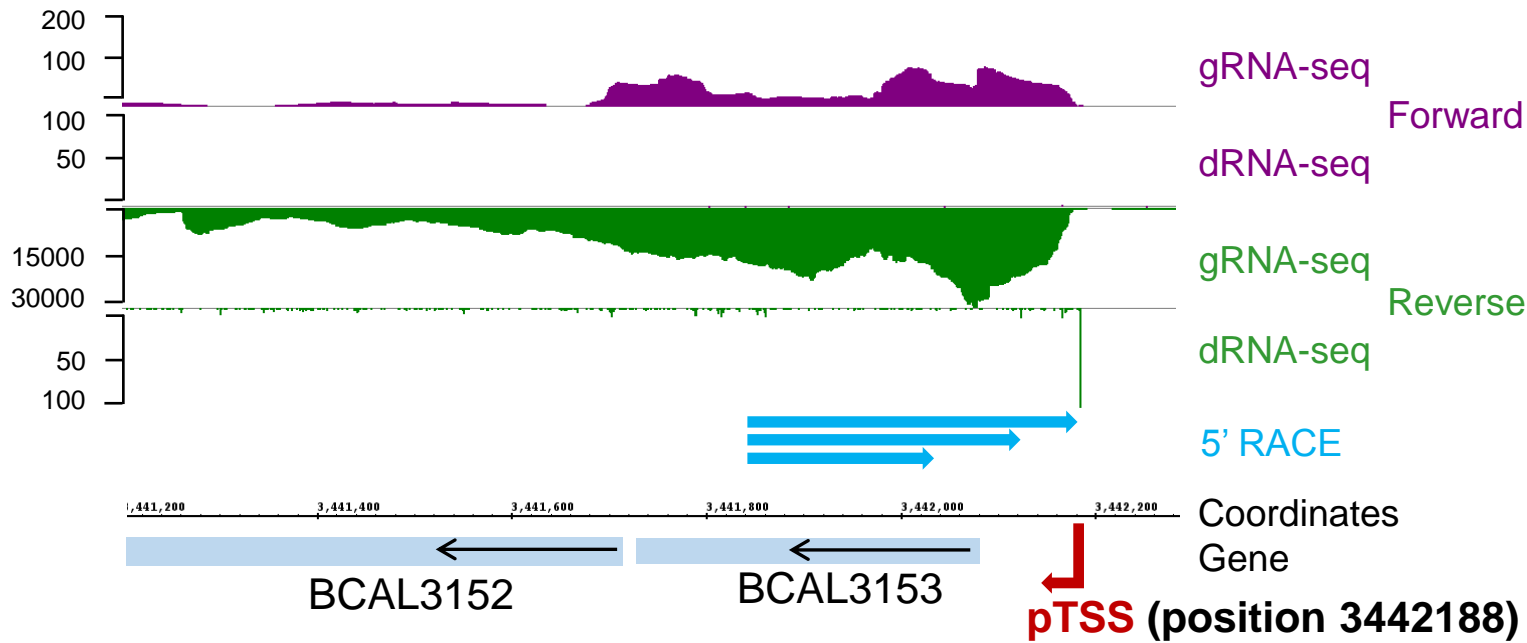

# Figure S1B

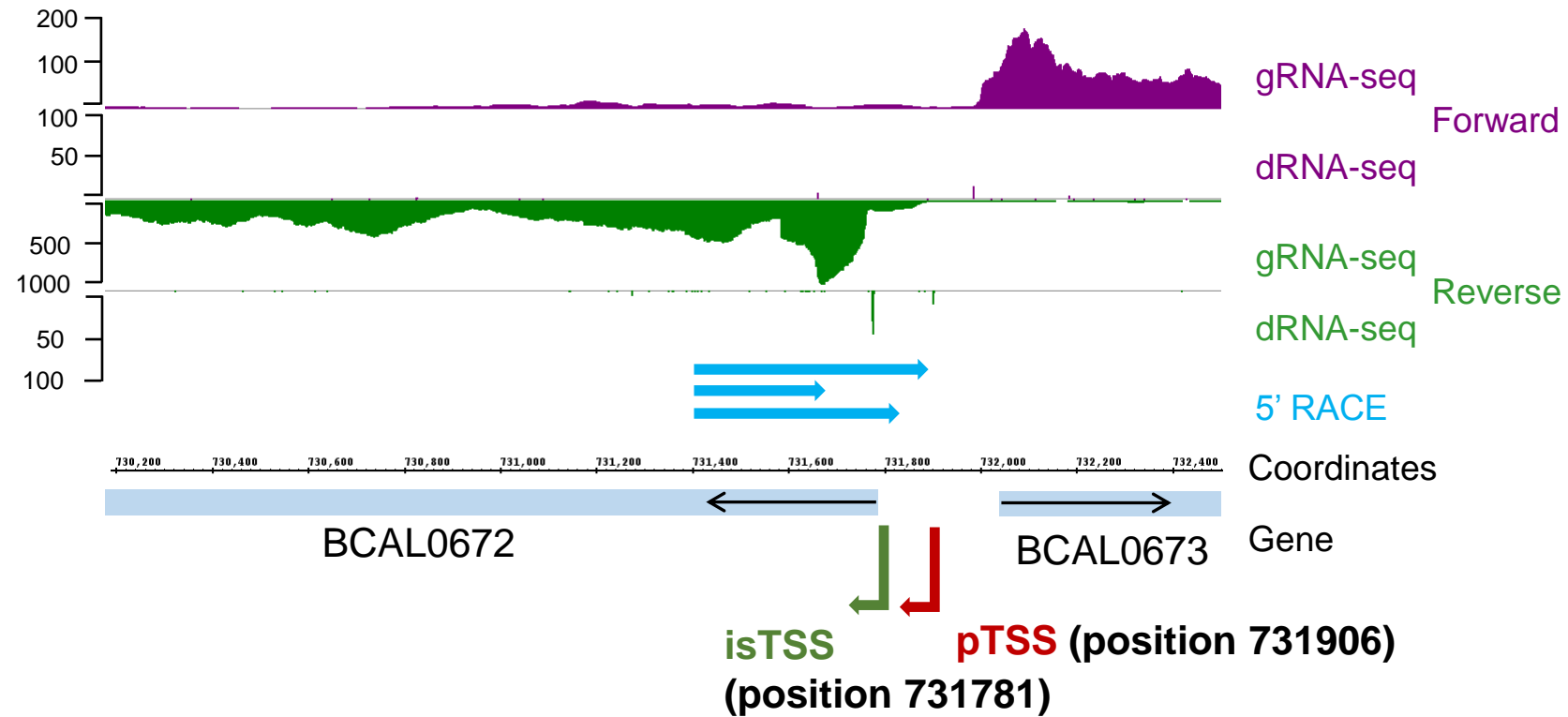

# Figure S1C

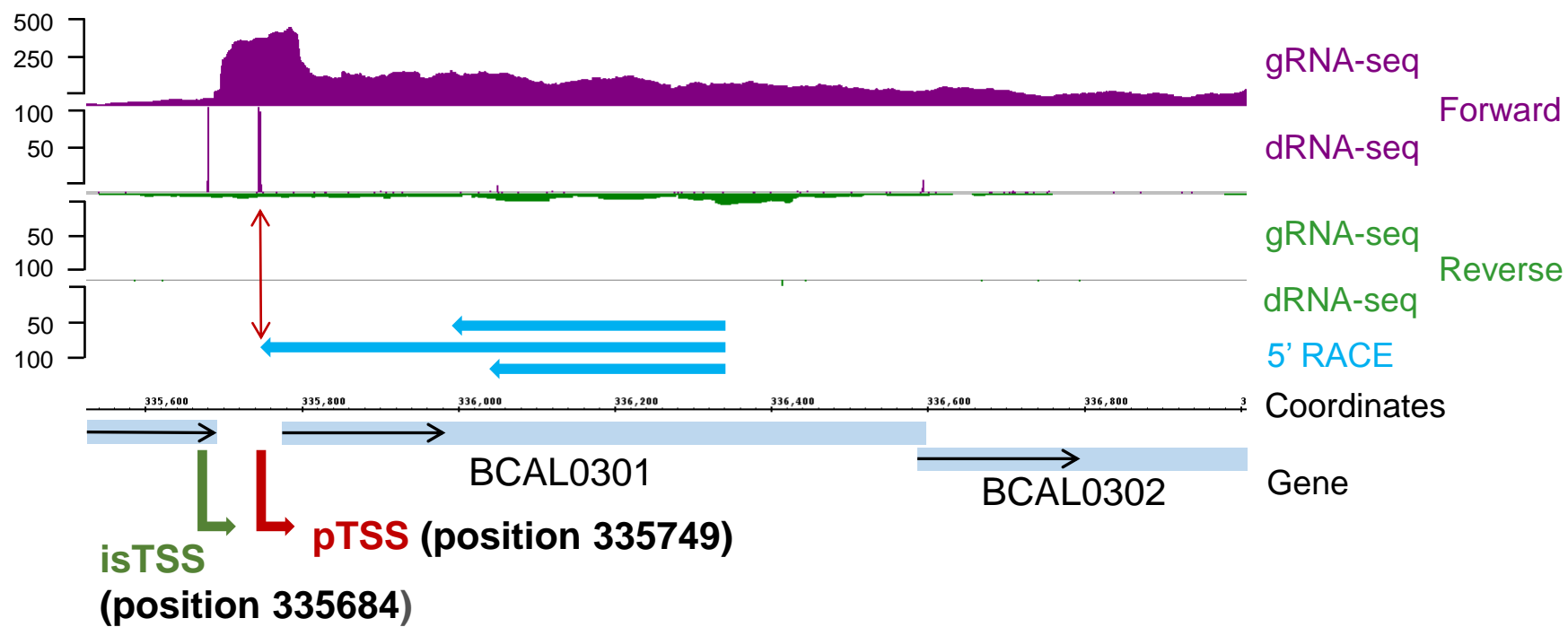

**Figure S1D**

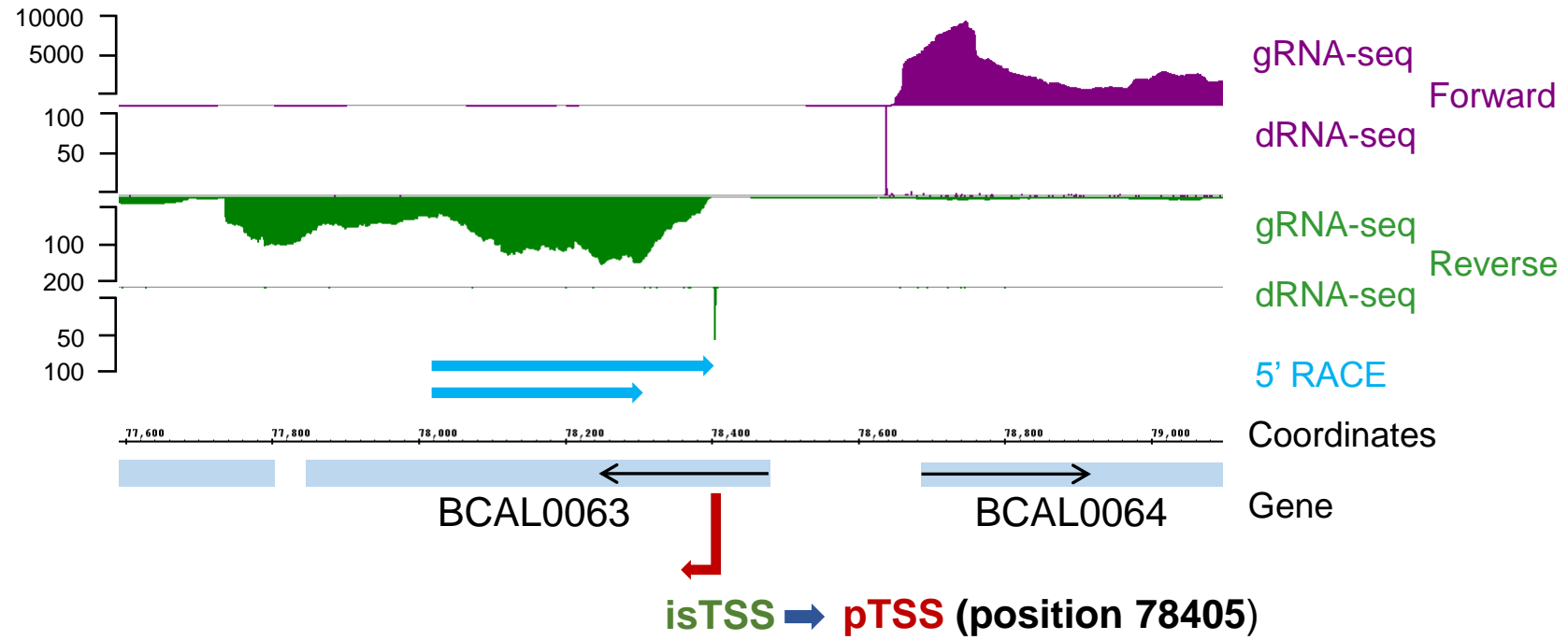

# Figure S1E

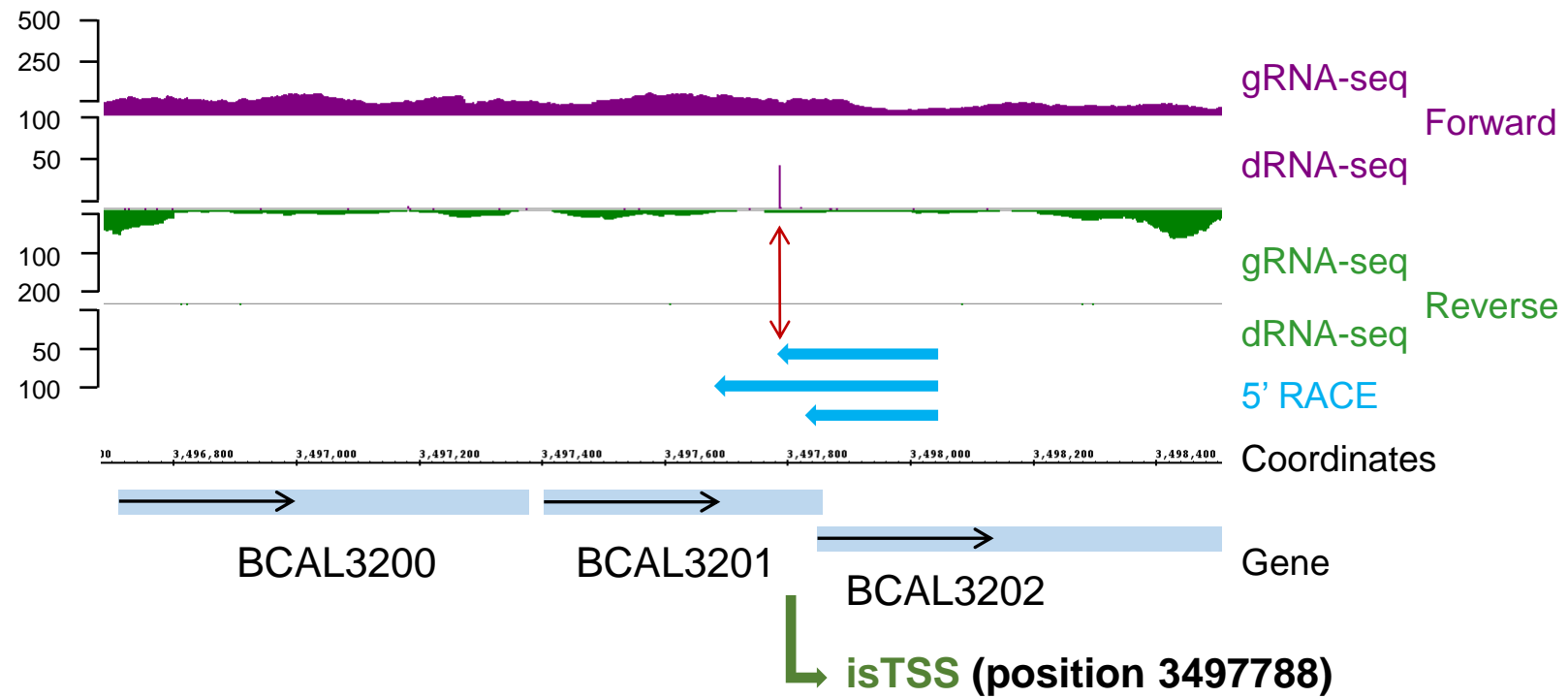

# Figure S1F

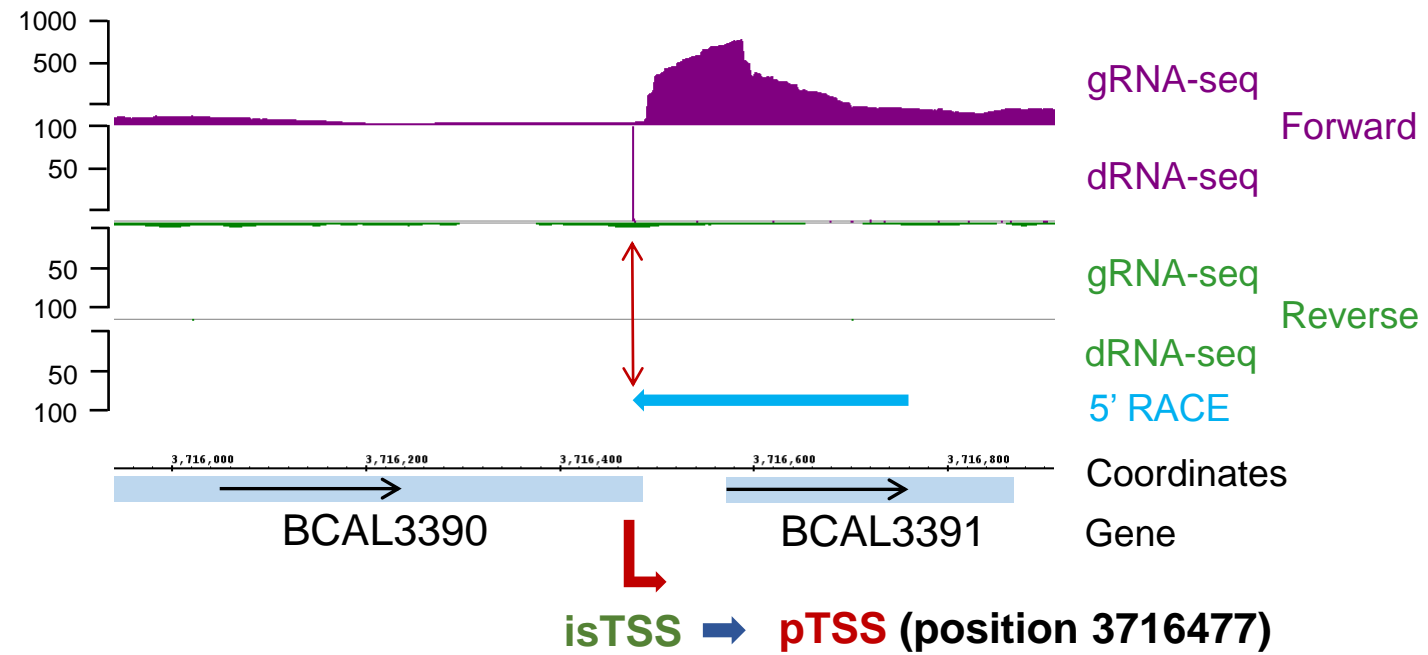

Supplement: Additional file 4: Figure S1. — Transcription start sites confirmed by 5′RACE. dRNA-Seq data are represented as number of read starts per base and gRNA-Seq data as read coverage, both are visualised using the Integrated Genome Browser [51]. dRNA-Seq data are represented as read starts per base, gRNA-Seq data are represented as coverage. Blue arrows depict representative sequences derived from 5′RACE analysis. Panel A: pTSS of BCAL3153, confirmed by 2 out of 8 RACE sequences, 6 sequences were shorter than the putative 5′UTR. Panel B: pTSS of BCAL0672, confirmed by 5 out of 12 RACE sequences, 7 sequences were shorter than the putative 5′UTR. The isTSS internal to BCAL0672 was not confirmed by 5′RACE, transcripts originating from this locus are probably truncated. Panel C: pTSS of BCAL0301 confirmed by 2 out of 7 RACE sequences, 5 sequences were shorter than the putative 5′UTR. The isTSS located internal to BCAL0300 could not be confirmed, transcripts originating from this locus are probably truncated. Panel D: Internal pTSS of BCAL0063. 4 out of 5 RACE sequences confirmed the internal pTSS, one read was shorter. Panel E: isTSS within BCAL3201: 3 out of 9 RACE sequences confirmed the isTSS, 3 were shorter and 3 longer than the transcript originating at this TSS. Sequences were therefore derived from transcripts originating from this TSS as well as from TSS further upstream in the operon. Panel F: pTSS for BCAL3391, internal to BCAL3390: confirmed by 6 out of 6 RACE sequences. (PDF 114 kb) [file 12864_2015_1993_MOESM4_ESM.pdf]
