# Supplementary material for: Genome-wide transcription start site profiling in biofilm-grown Burkholderia cenocepacia J2315
Source: BMC Genomics. 2015 Oct 13;16:775. doi: 10.1186/s12864-015-1993-3 (PMC4603805; doi:10.1186/s12864-015-1993-3)
Supplement: Additional file 12: Table S11. — Oligonucleotides used in this study. (DOCX 17 kb) [file 12864_2015_1993_MOESM12_ESM.docx]

**Genome-wide transcription start site profiling in biofilm-grown *Burkholderia cenocepacia* J2315**

**Supplementary table S11: Oligonucleotides used in this study**

| **5’ RACE** |  |  |
| --- | --- | --- |
| Target and primer function | Sequence | Distance to TSS |
| BCAL0672-GSP1 | GCACACCGAATTGAAGAAGG | 389 |
| BCAL0672-GSP2 | GCGTCGTATTCGTCTTCCAG | 280 |
| BCAL0672-nested | ACCTCCACGCATTCCTTCAC | 259 |
| BCAL0063-GSP1 | ACGTTCCACATCGATTACCAG | 416 |
| BCAL0063-GSP2 | ACGAAATCGACGAACAGCAG | 304 |
| BCAL0063-nested | CGTGTTGAGCAGGTTGTTGC | 252 |
| BCAL0301-GSP1 | AGCGTGCGGATCAGGTTC | 664 |
| BCAL0301-GSP2 | GACACCTCGGACGGCATC | 532 |
| BCAL0301-nested | GAGGTCGCGGATCAGGTC | 467 |
| BCAL3153-GSP1 | ACTGCTTTTTGCCGTCGTG | 363 |
| BCAL3153-GSP2 | CGCGATCGAATACGTACAGC | 229 |
| BCAL3153-nested | ACAGCGTCATGTGGGCTTC | 215 |
| BCAL3202-GSP1 | GGCACTTCGGTCCACAACTC | 253 |
| BCAL3202-GSP2 | TAGAGGAACAGCGCAAGCAG | 187 |
| BCAL3391-GSP1 | CGCGAACATCACGATCAGC | 284 |
| BCAL3391-GSP2 | CAGGTAGGGCGTCAACCAAG | 146 |
| **Quantitative RT-PCR** |  |  |
| Name (=target) and function | Sequence | Product size |
| BCAL0026-F control gene | TATGAAGTGCTGGTCGATGG | 190 |
| BCAL0026-R | TCAGCACGAAATCGTAGTCG |  |
| BCAL0972-F control gene | TCTCGAAGGTCTGGCACGAG | 107 |
| BCAL0972-R | CGTGATGTCGTGCTTCATCG |  |
| BCAL2367-F control gene | ACCATTTCCGCAACAAGGAC | 144 |
| BCAL2367-R | TGAAATCGGCCATGTACTGC |  |
| BCAL0813-F control gene | AGCTCAATCCGGAAGTCGTG | 106 |
| BCAL0813-R | AGCTGCTGTTTCAGCGATCC |  |
| BCAM0918-F control gene | GAGATGAGCACCGATCACAC | 143 |
| BCAM0918-R | CCTTCGAGGAACGACTTCAG |  |
| BCAL1895-F control gene | CAGCGGGTACGGGTTTCTTC | 122 |
| BCAL1895-R | GTTCTGGCCGTTGTTGATGC |  |
| BCAL2553-F control gene | TGATCTGGGTGGTCAAGCTG | 139 |
| BCAL2553-R | TGCAGGTCAAAATCGTCGTC |  |
| BCAS0059-F control gene | ATGCGGAATTCCAACAGGAG | 107 |
| BCAS0059-R | GCCCTTGCTCGAATAGTTGG |  |
| BCAL2383-F test gene | CACGAACTGGCAGGACTACG | 175 |
| BCAL2383-R | GAGGTCGACCCAGAAGTTGC |  |
